# Genetic variation in the TLL1 gene is not associated with fibrosis in patients with metabolic associated fatty liver disease

**DOI:** 10.1371/journal.pone.0243590

**Published:** 2020-12-11

**Authors:** Ali Bayoumi, Ismail Jalil, Mayada Metwally, Leon A. Adams, Rocio Aller, Carmelo García-Monzón, María Teresa Arias-Loste, Luca Miele, Salvatore Petta, Antonio Craxì, Rocio Gallego-Durán, Janett Fischer, Thomas Berg, Liang Qiao, Christopher Liddle, Elisabetta Bugianesi, Manuel Romero-Gomez, Jacob George, Mohammed Eslam

**Affiliations:** 1 Storr Liver Centre, Westmead Institute for Medical Research, Westmead Hospital and University of Sydney, Sydney, NSW, Australia; 2 Medical School, Sir Charles Gairdner Hospital Unit, University of Western Australia, Nedlands, WA, Australia; 3 Gastroenterology Svo., Hospital Clinico Universitario de Valladolid, School of Medicine, Valladolid University, Valladolid, Spain; 4 Liver Research Unit, Instituto de Investigacion Sanitaria Princesa, University Hospital Santa Cristina, CIBERehd, Madrid, Spain; 5 Gastroenterology and Hepatology Department, Marqués de Valdecilla University Hospital, Santander, Spain; 6 Department of Internal Medicine, Catholic University of the Sacred Heart, Rome, Italy; 7 Section of Gastroenterology and Hepatology, PROMISE, University of Palermo, Palermo, Italy; 8 Virgen del Rocío University Hospital, Institute of Biomedicine of Seville, Sevilla, Spain; 9 Division of Hepatology, Department of Medicine II, Leipzig University Medical Center, Leipzig, Germany; 10 Division of Gastroenterology, Department of Medical Science, University of Turin, Turin, Italy; Medizinische Fakultat der RWTH Aachen, GERMANY

## Abstract

Metabolic associated fatty liver disease (MAFLD) is the most prevalent liver disease in Western nations, with high heritability. A recent study of Japanese patients with the disease suggested that TLL1 rs17047200 is associated with fibrosis; whether a similar association is observed in Caucasian patients with MAFLD is unknown. We investigated the association of the *TLL1* rs17047200 polymorphism with liver fibrosis in a cohort of Caucasian patients with MAFLD (n = 728). We also investigated whether TLL1 expression is altered during liver injury in humans, in murine models of fibrosis, and in *in-vitro*. While TLL1 expression is upregulated in the liver of humans with MAFLD and in mice, the rs17047200 variant was not associated with fibrosis or any other histological features, or with hepatic TLL1 expression. In conclusion, the *TLL1* rs17047200 variant is not a risk variant for fibrosis in Caucasian patients with MAFLD. However, TLL1 could be involved in the pathogenesis of liver fibrosis.

## Introduction

To more accurately reflect disease the pathophysiology of fatty liver disease associated with metabolic dysfunction, the term non-alcoholic fatty liver disease (NAFLD) has recently been updated to metabolic (dysfunction)-associated fatty liver disease (MAFLD) [1–9]. MAFLD is a leading cause of end-stage liver disease, hepatocellular carcinoma (HCC) and liver transplantation, affecting 20–30% of the world’s population [3,10,11]. Despite its high prevalence, only a small proportion of patients progress to significant fibrosis, the major determinant of all-cause and liver-related mortality [12,13]. Thus, similar to other common diseases, MAFLD is a complex trait in which disease phenotype results from the dynamic interaction of environmental variables acting on a susceptible polygenic host background [14,15].

The heritable component of hepatic fibrosis has been estimated at ~50% based on a prospective twin study [16]. To date, the known risk polymorphisms explain only a small fraction of this heritability [14,17]. Thus, other as yet unidentified genetic and epigenetic variations likely exist that contribute to fibrosis risk. Notably, we and others have demonstrated that the fibrosis process shares common pathways across different liver diseases, but also has disease specific signals [18–24].

A recent genome-wide study in Japanese patients with chronic hepatitis C identified the *TLL1* rs17047200 single nucleotide polymorphism (or other intronic variants in strong LD), as a novel risk variant for HCC, indirectly via regulating liver fibrosis [25]. Consistently, we demonstrated in a large cohort of patients with chronic hepatitis C that rs17047200 is associated with fibrosis progression, but not fibrosis stage [26]. A recent study of 258 Japanese patients with biopsy-proven MAFLD has suggested that TLL1 rs17047200 is associated with fibrosis [27].

To increase our knowledge of shared genetic determinants influencing MAFLD and MAFLD-related fibrosis, we determined whether the rs17047200 polymorphism influences histological damage (fibrosis as the main objective) in a cohort of Caucasian patients (n = 728). We also investigated whether TLL1 expression is regulated during fibrosis in humans, in murine models of fibrosis, and *in-vitro*.

## Methods

### Patient cohort

The study comprised 728 European decent patients with biopsy-proven MAFLD, recruited from the participating centres between 2000–2015. Subjects with secondary causes of steatosis or alternative diagnoses were excluded, including alcohol abuse (men >30 g/day; women >20 g/day), total parenteral nutrition, chronic viral hepatitis (hepatitis B and hepatitis C), autoimmune liver disease, hereditary hemochromatosis, α1-antitrypsin deficiency, Wilson’s disease and drug-induced liver injury.

Ethics approval was obtained from the Human Research Ethics Committees of the Western Sydney Local Health District and the University of Sydney. All other sites had ethics approval from their respective ethics committees. Written informed consent for genetic testing was obtained from all participants.

### Clinical and laboratory assessment

Demographic and clinical data were obtained including age, gender, ethnicity, height and weight. Body mass index (BMI) was calculated as weight divided by the square of the height (kg/m^2^). Diabetes was defined as fasting blood glucose ≥ 7.0 mmol/L, previous diagnosis of diabetes or the use of antidiabetic drugs. The homeostasis model assessment (HOMA-IR) was calculated as: (fasting serum insulin [μU/mL] × fasting serum glucose [mmol/L])/22.5 [28]. At the time of liver biopsy, a fasting blood sample was obtained and routine biochemical tests were performed. Additional blood samples were drawn and frozen at −80°C for future research.

### Genotyping

Genotyping for *TLL1 rs17047200* was undertaken using the TaqMan SNP genotyping allelic discrimination method (Applied Biosystems, Foster City, CA, USA). All genotyping was blinded to clinical variables.

### Liver histopathology

Liver biopsies were scored by an expert liver pathologist in each participating centre unaware of the clinical or genetic data. Histological scoring was based on the system proposed by Kleiner et al.^29^ Steatosis was graded from 0 to 3, lobular inflammation from 0 to 3 and hepatocellular ballooning from 0 to 2. Fibrosis was staged from 0 to 4 with 4 representing cirrhosis. The steatohepatitis activity score (NAS) was calculated to quantify disease activity [29]. The inter-observer agreement between pathologists was studied previously and was excellent for steatosis (κ = 0.85) and good for fibrosis (κ = 0.78) [30].

### TLL1 gene expression in human tissues, human primary hepatic cells and human MAFLD

Real-time quantitative polymerase chain reaction was used to assess TLL1 mRNA expression in a selection of 18 human tissues using TissueScan Human Normal cDNA Array (Origene, Rockville, MD). TLL1 mRNA levels were assessed in human primary hepatocytes, primary human hepatic stellate cells and primary human hepatic sinusoidal endothelial cells (ScienceCell, Carlsbad, CA) and in available tissue samples from patients with steatohepatitis (n = 24). Control samples were from patients undergoing resection for benign liver tumours in whom all causes of liver disease were excluded (n = 27). Steatohepatitis was diagnosed using standard histological criteria, as follows^29^: samples with a steatohepatitis activity score (NAS) of ≥ 5 or a NAS of 3–4 but with fibrosis. mRNA expression levels were determined using TaqMan probes and master mix (Life Technologies) according to the manufacturer’s protocol. Gene expression levels were normalized for GAPDH.

### Animal experimental protocol

Three different models of fibrosis were employed as generated by SMC Laboratories, Inc. (Japan). Five mice from each fibrosis model and five mice from each control group were included in this study. For carbon tetrachloride (CCl4)-induced fibrosis, male C57BL/6 mice were injected intraperitoneally with CCl4 (100 μl of 5% CCl4 in mineral oil/twice weekly) for 4 weeks to induce liver fibrosis. Control mice received an equivalent volume of mineral oil. Bile duct ligation or sham ligation was for two weeks. Experimental fibrosing steatohepatitis was induced by administration of a methionine and choline-deficient diet (MCD diet) or a control diet supplemented with methionine and choline for 8 weeks.

### SMC Laboratories animal welfare standard operating procedures (SOPs)

The animals were maintained in a SPF facility under controlled conditions of temperature (23 ± 2°C), humidity (45 ± 10%), lighting (12-hour artificial light and dark cycles; light from 8:00 to 20:00) and air exchange. The animals were housed in TPX cages that were appropriate in size for the number of mice (according to the recommendations set in the Guide for the Care and Use of Laboratory Animals (8th Ed) _ILAR). Sterilized paper bedding and nesting material were used and replaced once a week. Sterilized solid diet was placed in a metal lid on the top of the cage. Pure water was provided from a water bottle equipped with a rubber stopper and a sipper tube. Water bottles were replaced once a week, cleaned, and sterilized in an autoclave and reused. If animals showed clinical signs of morbidity such as prone position, the animal was euthanized by under excess isoflurane. The viability, clinical signs (e.g., lethargy, twitching, labored breathing) and behavior were monitored daily.

### Culture of human HSCs

Human HSCs (LX2) and primary human HSCs (ScienCell Research Laboratories (San Diego, CA, USA)) were treated with TGFβ-1 (5ng/ml) for 24 hours and then evaluated for mRNA expression.

All cell lines are regularly tested for mycoplasma contamination. Additionally, regular short tandem repeat (STR) profiling is undertaken for authentication.

### Statistical methods

Results are expressed as mean ± SD (standard deviation), median and interquartile range or number (percentage) of patients. The Student’s *t*-test or non-parametric, i.e. Wilcoxon-Mann-Whitney U-test or Kruskal-Wallis tests were used to compare quantitative data as appropriate. χ^2^ and Fisher-exact tests were used for comparison of frequency data and to evaluate the relationships between groups. The Cochran–Armitage test was used for trend analysis. All tests were two-tailed and p values <0.05 were considered significant. Genetic analyses were performed assuming a dominant model of inheritance due to the low frequency of minor homozygotes, as previously published [25,26].

Multiple logistic regression models were fitted to binary traits incorporating biologically relevant covariates associated with the risk of liver fibrosis (age, gender, Body Mass Index (BMI), presence of type 2 diabetes mellitus (T2DM), recruiting centre). Steatosis was dichotomized as grade 1 or Grade 2 and 3, inflammation was dichotomized as absent/mild (A0-A1) or moderate/severe (A2-A3) and fibrosis as absent/mild (F0-1) or significant (F2-4) or advanced fibrosis (F0-F2) vs (F3-F4). Results are expressed as odds ratios and 95% confidence intervals (CI). A power analysis was performed using the G*power program (http://www.gpower.hhu.de/) [31]. This confirmed that our cohort had > 95% power to detect an effect size index of 0.2. Statistical analyses were performed using the statistical software package SPSS for Windows, version 21 (SPSS, Chicago, IL) or program R.

## Results

### The TLL1 rs17047200 is not associated with steatosis or histological disease severity in MAFLD

The clinical and biochemical characteristics of the cohort are summarized in **Table 1** and are representative of the general population. *TLL1* rs17047200 was confirmed to be in Hardy–Weinberg equilibrium with a minor allele frequency of 0.15, similar to that observed in other European populations (MAF = 0.15), http://browser.1000genomes.org).

**Table 1:** Demographic, anthropometric and clinical characteristics of the patient cohort with MAFLD.

Variables	MAFLD cohort (n = 728)
**Age (yrs)**	47 (37.3–56.9)
**Male (%)**	381 (52.3)
**BMI (Kg/m**^2^)	31 (27.2–39.9)
**ALT (IU/L)**	47 (25–77)
**AST (IU/L)**	31 (20–45)
**Platelet (x10**^**9**^**/L)**	250 (201–297)
**Cholesterol (mmol/L)**	4.84 (4.22–5.67)
**Triglycerides (mmol/L)**	1.43 (1.04–2.1)
**HDL-C (mmol/L)**	1.25 (1.02–1.56)
**LDL-C (mmol/L)**	2.82 (2.3–3.53)
**Blood glucose (mmol/L)**	5.4 (4.9–6.2)
**HOMA-IR**	3.03 (1.87–4.66)
**Steatosis grade** 2–3 (%)	269 (37)
**Lobular inflammation** 2–3 (%)	195 (26)
**Fibrosis Stage** 2–4 (%)	229 (31.5)
**Fibrosis Stage** 0, 1, 2, 3, 4 (%)	241(33.1), 258(35.4), 150(20.6), 48(6.6), 31(4.3)
***TLL1* rs17047200** AA, AT, TT (%)	527 (72.4), 181 (24.9), 20 (2.7)

Values are median with interquartile range or frequency and percentage. P values for Hardy-Weinberg equilibrium were calculated by chi square test and was p = 0.3. P >0.05 indicates no deviation from Hardy-Weinberg equilibrium.

**Fig 1:**
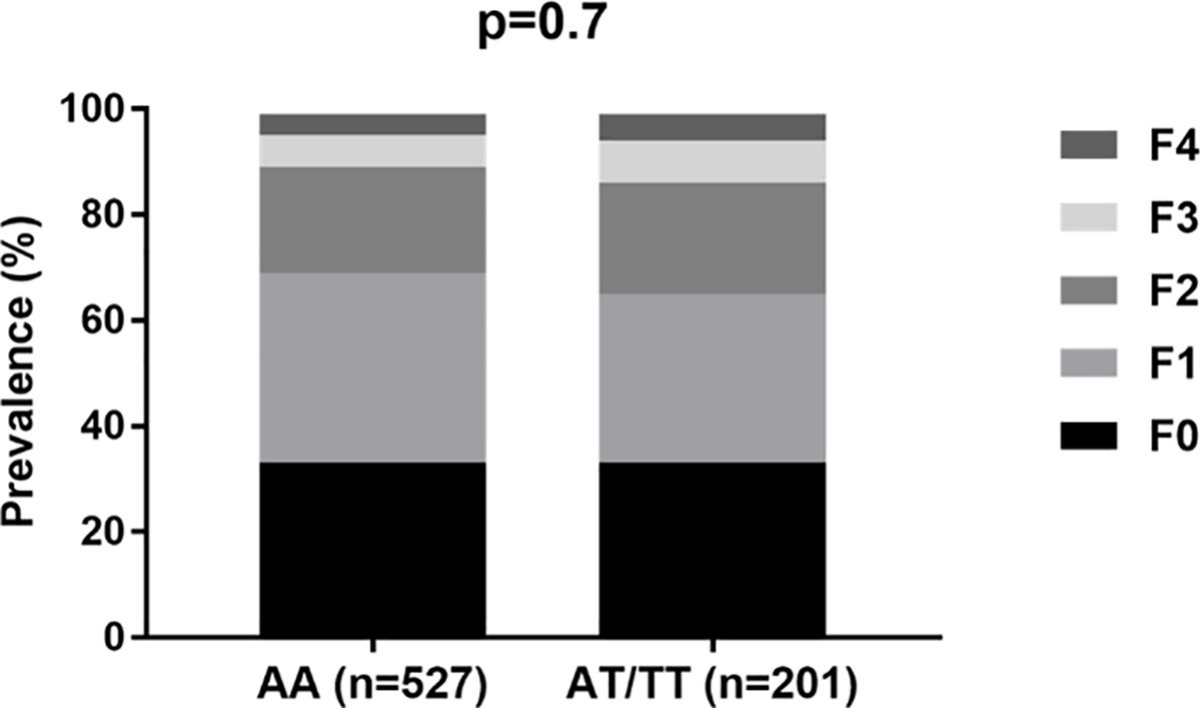
Frequencies of TLL1 genotypes according to hepatic fibrosis stage.

The association analysis of rs17047200 to MAFLD showed no relationship with fibrosis (p  =  0.7) using a dominant model, as previously reported (**Fig 1, S1 Table**). Likewise, no association was observed when analysis was undertaken subdividing the cohort into those with mild (F0–1) versus significant fibrosis (F2–4) (p = 0.3) or into early (F0-F2) vs advanced fibrosis (F3-F4) (p = 0.2). The lack of association remained unaltered after adjusting for age, sex, BMI, and ALT (OR: 1.2, 95%:0.828–1.745, p = 0.3). No associations were observed between rs17047200 and any other histological features, including steatosis (p = 0.3), lobular inflammation (p  =  0.8) or the NAS score (p  =  0.2). Similarly, we found no correlation between rs17047200 and any clinical variables (**S2 Table**).

### TLL1 is expressed in liver and in hepatic stellate cells

**Fig 2:**
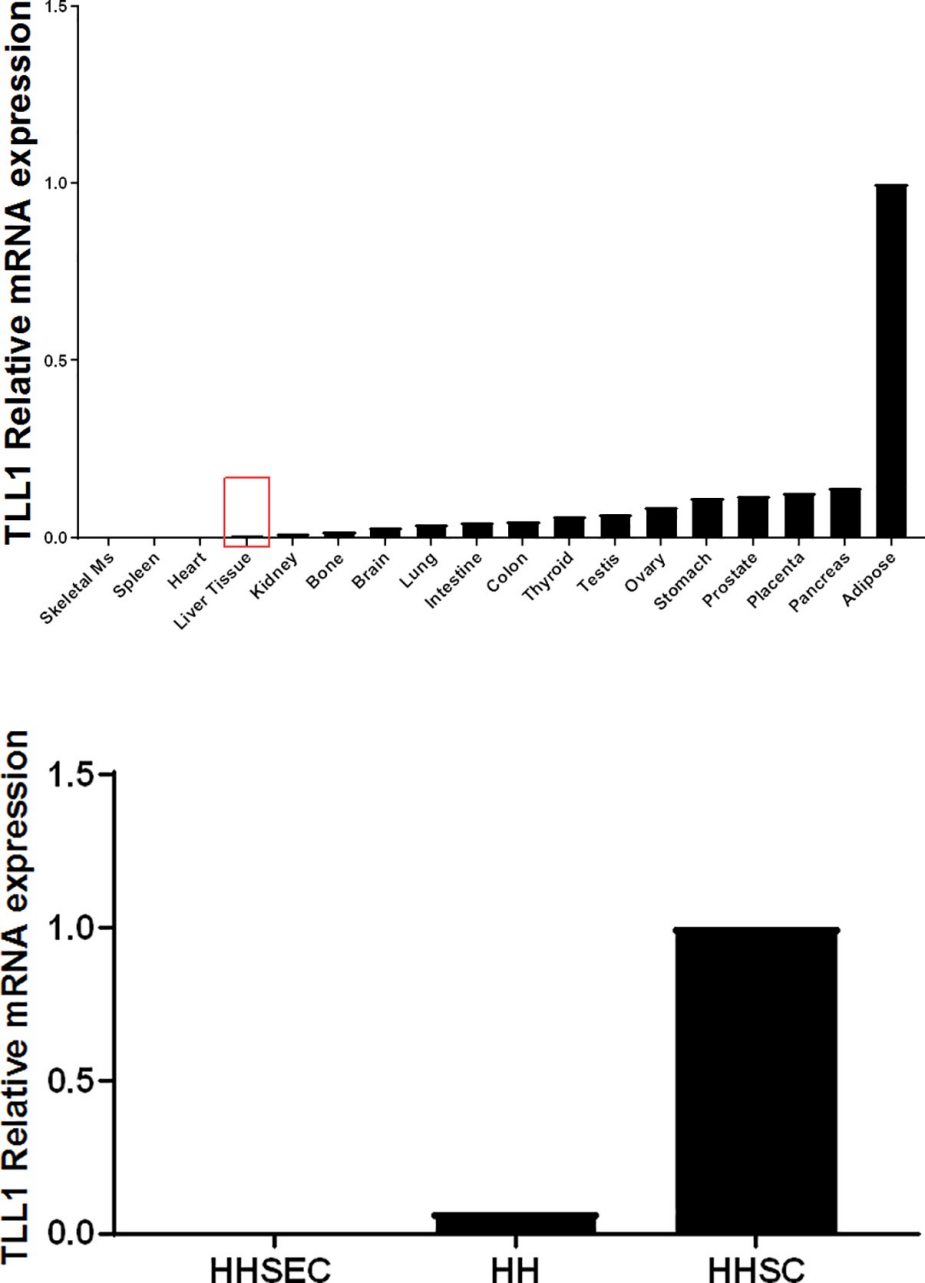
TLL1 expression in human tissues and primary human hepatic cells (A) Distribution of TLL1 mRNA expression in 18 human tissues. (B) TLL1 mRNA expression in primary human hepatic cell types. Gene expression levels were assessed by quantitative polymerase chain reaction. The tissue or cell line with the highest CT value was assigned a value of 1. HH, human hepatocytes; HHSEC, human hepatic sinusoidal endothelial cell; HHSC, human hepatic stellate cell.

To investigate if TLL1 expression is related to fibrosis, we first examined mRNA expression of the gene in several human tissues. TLL1 was expressed in many tissues including the liver, though expression was low (**Fig 2A**). We next examined TLL1 expression in primary human hepatocytes, human stellate cells, and hepatic sinusoidal endothelial cells. Overall, TLLI mRNA expression levels were low in all three cell types, with highest expression in hepatic stellate cells (HHSC) suggesting a potential role in fibrosis (**Fig 2B**). Given this result, we investigated the expression of *TLL1* during fibrosis in *in vitro* and *in vivo* models, as well as in human samples.

### Hepatic expression of TLL1 in patients with MAFLD

**Fig 3:**
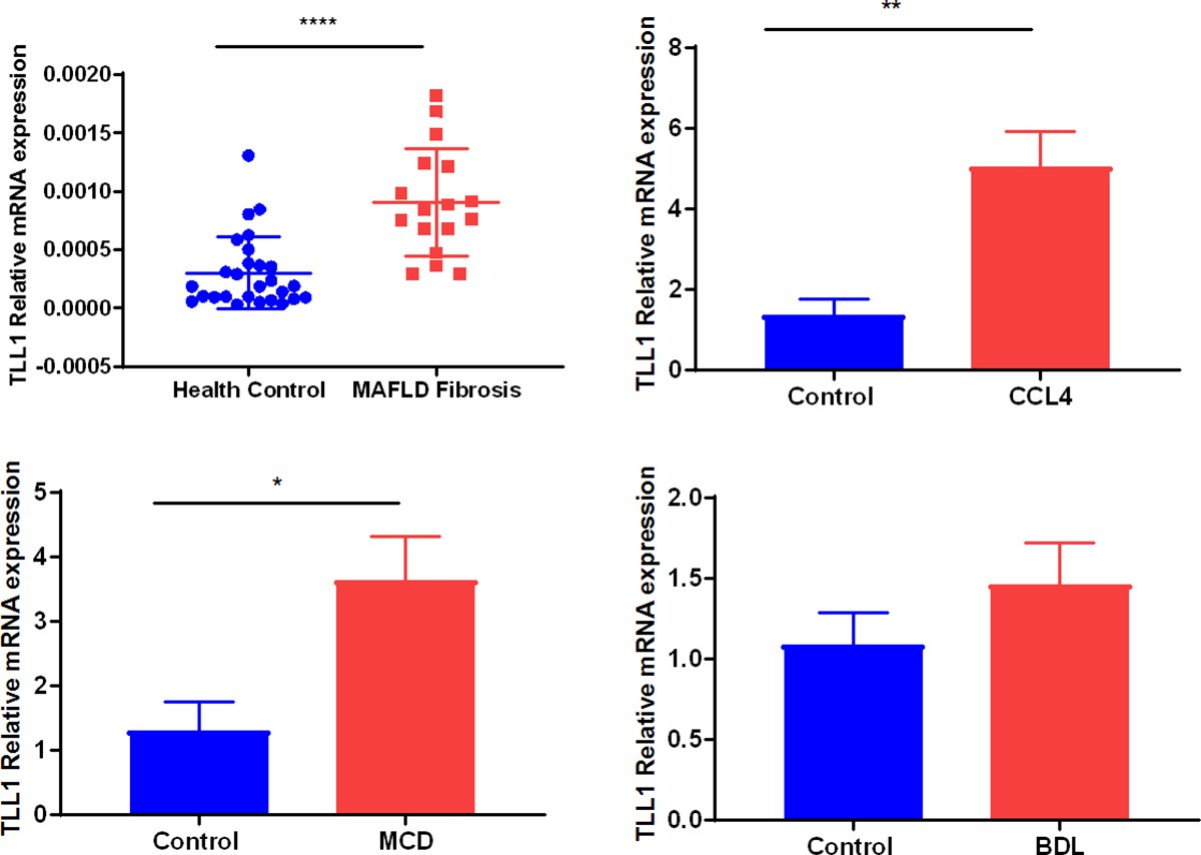
Hepatic TLL1 expression in patients with MAFLD and control subjects, and in mice models of fibrosis. The relative expression levels of TLL1 in liver samples obtained from non-steatotic controls (n = 27) and subjects with metabolic steatohepatitis (n = 17) (A). qPCR analysis for TLL1 expression in liver samples from three well-established models of fibrosis in the mouse, namely carbon tetrachloride (CCl4) administration (B), bile duct ligation (BDL) (C) and, feeding a diet deficient in methionine and choline (MCD) (D). Gene expression was normalized to that of GAPDH.

To investigate the expression of TLL1 in human liver from patients with metabolic steatohepatitis and control subjects, we assessed 17 steatohepatitis patients, their characteristics are depicted in **S3 Table** and 27 controls as described in methods. TLL1 mRNA levels were higher in livers from patients with steatohepatitis compared to that in normal liver (**Fig 3A**). However, the rs17047200 allele did not influence hepatic TLL1 expression.

### TLL1 mRNA expression is correlated with fibrosis in animal and in-vitro models of hepatic fibrosis

To investigate the potential role of TLL1 in fibrosis, we measured its expression in livers from mice subject to three models of fibrosing liver injury, namely carbon tetrachloride (CCl4) administration, bile duct ligation (BDL), and feeding a methionine-choline deficient (MCD) diet. The latter model is more representative of fibrosis associated with steatohepatitis. As shown in **Fig 3B–3D**, TLL1 expression was induced with fibrosis in all three models. *In vitro*, TLL1 expression was induced after treatment with recombinant TGF-β1 of both human stellate cells (LX2) and primary HSCs (**S1 Fig**).

Overall, these data provide additional support for a potential profibrogenic role for TLL1 but this effect is unlikely to be modulated by rs17047200.

## Discussion

Considering recent evidence that *TLL1* rs17047200 is a novel risk variant for fibrosis progression and liver cancer in patients with chronic hepatitis C [25,26], we examined its potential role in MAFLD. As we demonstrate, the *TLL1* rs17047200 variant did not influence any histological parameter (steatosis, inflammation or fibrosis) in our large cohort of Caucasian patients. However, TLL1 is expressed by human HSCs, is overexpressed in patients with metabolic steatohepatitis, and in murine and *in-vitro* models of fibrosis.

A recent small study of Japanese patients with MAFLD (n = 258) has suggested that the *TLL1* rs17047200 T allele is associated with advanced liver fibrosis (p = 0.044) [27]. The reason for our discrepant results is unclear, but might be attributed to differences in baseline characteristics, the frequencies of the *TLL1* genotype (MAF in our cohort was 0.15, while it was 0.10 in the Japanese cohort), variant size effects or differences in cohort size. The size effect of any SNP may also vary across different populations. It is unlikely that our data could have arisen as a result of a type 2 error (false-negative results) because initial power estimations demonstrated that our cohort had > 95% power to detect an effect size index of 0.2. No power analysis was provided in the other study [27]. In total, the findings suggest that the *TLL1* rs17047200 genetic variant is either not a common genetic marker for liver fibrosis across different etiologies or alternatively that rs17047200 is unlikely to be the causative variant in Caucasian patients. Further studies are thus required to clarify its true role.

We observed that TLL1 was expressed in many tissues including at a low level in whole liver. At a cellular level, TLL1 was most highly expressed in primary hepatic stellate cells. An intriguing finding was that hepatic TLL1 expression was higher in patients with steatohepatitis compared to healthy controls. Consistent with our genetic results, TLL1 expression was not different according to rs17047200 genotype. Further, increased expression of TLL1 was observed in three independent murine models of fibrosis, with the MCD diet model resembling human metabolic steatohepatitis. We also demonstrated that TLL1 is induced in cultured human HSC express in response to TGFβ stimulation. TLL1 is an astacin-like metalloprotease that shares structural similarity to bone morphogenetic protein-1 (BMP1) and specifically processes procollagen C-propeptides at the physiologically relevant site, whereas TLL2 lacks this activity [32]. This could explain why it is expressed mainly in HSCs in the liver given its role in collagen processing. Further studies in *in vivo* models are needed to define the role of TLL1 in the pathogenesis of fibrosis in general and during the development of metabolic steatohepatitis.

The strength of our study lies in the large number of patients enrolled. However, some limitations must be acknowledged, including the fact that given the cross-sectional nature of the study we could not dissect associations with fibrosis progression. We also cannot rule out the possibility that other variants in this or other genes in linkage disequilibrium with rs17047200 may be the functional variant. Notably, all SNPs even in weak LD with rs17047200 (LD = 0.20) in Europeans are intronic variants.

In conclusion, we demonstrate that the *TLL1* rs17047200 variant is not a risk variant for fibrosis severity in Caucasian patients with MAFLD. However, TLL1 is likely involved in the pathogenesis of liver fibrosis.

## Supporting information

S1 Fig Gene expression levels of TLL1in a human hepatic stellate cell line (LX2) (A) and in primary Human HSCs (B). Human HSCs (LX2) or primary human hepatic stellate cells were treated with human recombinant TGF-β1 (5 ng/mL) or mock-treated for 24 hours. The relative levels of TLL1 mRNA were normalized to control. Three independent experiments were carried out. Data represent mean ± SEM.
(DOCX)

S1 Table Association of TLL1 rs17047200 genotype with degree of fibrosis. (DOCX)

S2 Table Demographic, anthropometric and clinical characteristics of the cohort stratified according to *TLL1* rs17047200 genotype. (DOCX)

S3 Table Clinical Characteristics of MAFLD patients for whom TLL1 hepatic mRNA levels evaluation was available. (DOCX)
