# Supplementary material for: Genetic variation in the TLL1 gene is not associated with fibrosis in patients with metabolic associated fatty liver disease
Source: PLoS One. 2020 Dec 11;15(12):e0243590. doi: 10.1371/journal.pone.0243590 (PMC7732106; doi:10.1371/journal.pone.0243590)
Supplement: S1 Fig — Gene expression levels of TLL1in a human hepatic stellate cell line (LX2) (A) and in primary Human HSCs (B). Human HSCs (LX2) or primary human hepatic stellate cells were treated with human recombinant TGF-β1 (5 ng/mL) or mock-treated for 24 hours. The relative levels of TLL1 mRNA were normalized to control. Three independent experiments were carried out. Data represent mean ± SEM. (DOCX) [file pone.0243590.s001.docx]

**A B**


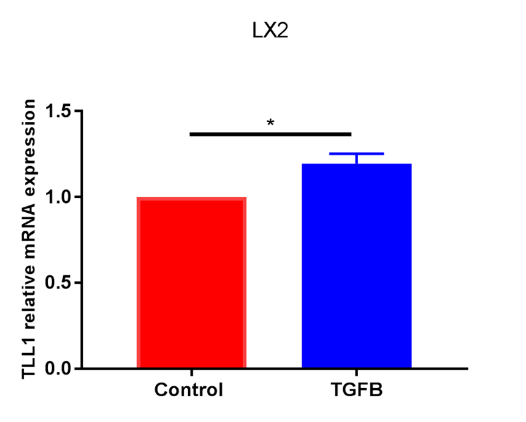

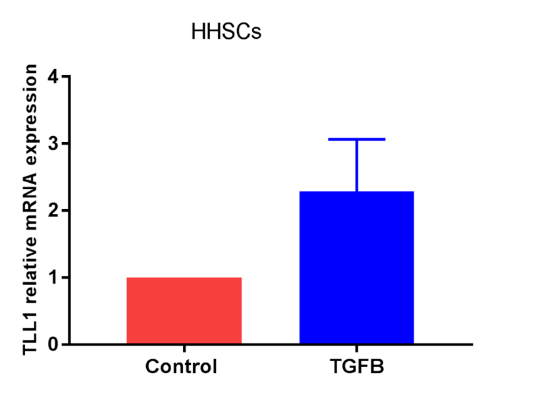


**Supplementary figure 1:** Gene expression levels of TLL1in a human hepatic stellate cell line (LX2) (A) and in primary Human HSCs (B). Human HSCs (LX2) or primary human hepatic stellate cells were treated with human recombinant TGF-β1 (5 ng/mL) or mock-treated for 24 hours. The relative levels of TLL1 mRNA were normalized to control. Three independent experiments were carried out. Data represent mean ± SEM.
