# Supplementary material for: Genetic variation in the TLL1 gene is not associated with fibrosis in patients with metabolic associated fatty liver disease
Source: PLoS One. 2020 Dec 11;15(12):e0243590. doi: 10.1371/journal.pone.0243590 (PMC7732106; doi:10.1371/journal.pone.0243590)
Supplement: S1 Table — (DOCX) [file pone.0243590.s002.docx]

**Table 1: Association of TLL1 rs17047200 genotype with degree of fibrosis**

| ***TLL1* rs17047200** | **Fibrosis Degree** | | | | |
| --- | --- | --- | --- | --- | --- |
|  | **F0** | **F1** | **F2** | **F3** | **F4** |
| AA | 174 (33) | 193 (36.6) | 107 (20.3) | 32 (6.1) | 21 (4.0) |
| AT | 60 (33.1) | 57 (31.5) | 39 (21.5) | 15 (8.3) | 10 (5.5) |
| TT | 7 (35.0) | 8 (40.0) | 4 (20.0) | 1 (5.0) | 0 (0.0) |
