# Supplementary material for: Genetic variation in the TLL1 gene is not associated with fibrosis in patients with metabolic associated fatty liver disease
Source: PLoS One. 2020 Dec 11;15(12):e0243590. doi: 10.1371/journal.pone.0243590 (PMC7732106; doi:10.1371/journal.pone.0243590)
Supplement: S2 Table — (DOCX) [file pone.0243590.s003.docx]

**Table 2: Demographic, anthropometric and clinical characteristics of the cohort stratified according to *TLL1* rs17047200 genotype**

| **Variables** | **AA (n=527)** | **AT/TT (n=201)** | **p-value** |
| --- | --- | --- | --- |
| **Age (yrs)** | 47(37.7-57) | 46(33-56) | 0.4 |
| **Male (%)** | 272 (52) | 109 (54) | 0.5 |
| **BMI (Kg/m**^2^) | 30.8(27.2-38.7) | 31.8(27.6-41.4) | 0.1 |
| **ALT (IU/L)** | 47(25-77) | 46 (24-77) | 0.9 |
| **AST (IU/L)** | 31(20-45) | 30 (20-48) | 0.8 |
| **Platelet (x10^9^/L)** | 250 (200-299) | 249 (202-289) | 0.7 |
| **Cholesterol (mmol/L)** | 4.8 (4.2-5.7) | 4.73 (4.12-5.59) | 0.3 |
| **Triglycerides (mmol/L)** | 1.39 (1.04-2.1) | 1.54 (1.05-2.11) | 0.1 |
| **HDL-C (mmol/L)** | 1.25 (1.02-1.58) | 1.23 (1.02-1.50) | 0.2 |
| **LDL-C (mmol/L)** | 2.82 (2.3-3.59) | 2.84 (2.17-3.51) | 0.4 |
| **Blood glucose (mmol/L)** | 5.5 (4.9-6.2) | 5.3 (4.8-6.4) | 0.2 |
| **HOMA-IR** | 3.1 (1.9-4.8) | 2.97 (1.87-4.59) | 0.7 |
