# Supplementary material for: Genetic variation in the TLL1 gene is not associated with fibrosis in patients with metabolic associated fatty liver disease
Source: PLoS One. 2020 Dec 11;15(12):e0243590. doi: 10.1371/journal.pone.0243590 (PMC7732106; doi:10.1371/journal.pone.0243590)
Supplement: S3 Table — (DOCX) [file pone.0243590.s004.docx]

**Table 3: Clinical Characteristics of MAFLD patients for whom TLL1 hepatic mRNA levels evaluation was available**

| **Variables** |  |
| --- | --- |
| **Age (yrs)** | 51.7 ± 13.96 |
| **Male (%)** | 13 (76.4) |
| **BMI (Kg/m**^2^) | 31.43 ± 5.7 |
| **ALT (IU/L)** | 78.9 ± 40.4 |
| **AST (IU/L)** | 56.4 ± 25.52 |
| **Platelet (x10^9^/L)** | 212.3 ± 67.34 |
| **HOMA-IR** | 7.9 ± 6.57 |
| **Blood glucose (mmol/L)** | 8.2 ± 3.82 |
| **Cholesterol (mmol/L)** | 5.1 ± 1.43 |
| **Triglycerides (mmol/L)** | 2 ± 1.15 |
| **HDL-C (mmol/L)** | 1.1 ± 0.33 |
| **LDL-C (mmol/L)** | 2.6 ± 1.05 |
